# Longitudinal Variability of Fecal Calprotectin in Preterm Newborns: A Prospective Cohort Study

**DOI:** 10.3390/pediatric18040087

**Published:** 2026-07-01

**Authors:** Mariana A. Polimeni Cavassin Jayme, Cristina Terumi Okamoto, Fernanda Tiemi Takei, Paula Haus de Oliveira, Eloisa Medeiros Nisihara, Renato Nisihara

**Affiliations:** 1 Clinical Hospital, Federal University of Paraná, Curitiba 80320-300, Paraná, Brazil; marianapolimeni@gmail.com; 2 Department of Medicine, Mackenzie Evangelical School of Medicine of Paraná, Curitiba 80730-000, Paraná, Brazil; cristoka0810@gmail.com (C.T.O.); fertakei@gmail.com (F.T.T.); eloestudoss@gmail.com (E.M.N.); 3 Department of Medicine, Positivo University, Curitiba 81380-330, Paraná, Brazil; paulahaus.de@gmail.com; 4 Neonatal Intensive Care Unit, Hospital do Trabalhador, Curitiba 81050-000, Paraná, Brazil

**Keywords:** gastrointestinal inflammation, fecal calprotectin, preterm

## Abstract

Fecal calprotectin (FC) is a potential biomarker of gastrointestinal inflammation; however, its physiological behavior in preterm newborns remains poorly understood. This prospective cohort study aimed to characterize the longitudinal variability of FC concentrations during the first month of life in preterm newborns of ≤34 weeks of gestational age admitted to a neonatal intensive care unit. Altogether, 48 preterm newborns and 42 mothers were examined, with 124 fecal samples collected weekly. The median FC levels exhibited wide interindividual and intraindividual variations, ranging from 56 µg/g in the first week to 65 µg/g in the third week, with no significant association with clinical or laboratory variables. No confirmed cases of NEC occurred during follow-up. Among the five preterm newborns with clinical suspicion of NEC, FC levels fluctuated without a consistent temporal pattern or discriminatory profile. Because stool samples were collected according to a predefined weekly schedule rather than at symptom onset, transient FC changes associated with acute gastrointestinal events may not have been captured. The very small number of newborns with clinically suspected NEC, particularly during later follow-up, substantially limited the statistical power of subgroup analyses. Therefore, statistical comparisons involving this subgroup should be interpreted as exploratory and hypothesis-generating rather than confirmatory. Therefore, FC levels may vary substantially in preterm newborns and, within the limitations of this study, these findings primarily characterize the baseline longitudinal variability of FC rather than its diagnostic value for NEC and support cautious interpretation of isolated FC measurements in this population.

## 1. Introduction

Necrotizing enterocolitis (NEC) is the major cause of gastrointestinal-related death in preterm newborns, affecting 5–12% of very low-birth-weight newborns [1]. The pathophysiology of NEC is complex and multifactorial. Primary risk factors include prenatal conditions such as chorioamnionitis and genetic imprinting; perinatal factors such as low gestational age, low birth weight, and abnormal gut microbiota and neonatal care-related factors such as mechanical ventilation, type of feeding, and pharmacological interventions [2]. Review studies have demonstrated that NEC is associated with high mortality rates and affects neurodevelopment, specifically in developed countries, although these studies provide limited information on broader aspects of quality of life [2,3]. The limited understanding of the pathogenesis, coupled with nonspecific initial clinical symptoms, causes diagnostic delays and represents a notable challenge in identifying specific biomarkers for NEC. Early detection using biomarkers may help prevent severe consequences and improve understanding of the disease. Nevertheless, specific molecular biomarkers for NEC have yet to be identified [4].

Although fecal calprotectin (FC) has emerged as one of the most extensively investigated noninvasive biomarkers for NEC, considerable uncertainty remains regarding its physiological variability during early neonatal life. This biological variability complicates the interpretation of isolated FC measurements and limits their routine clinical applicability in preterm infants. Some authors reported increased FC concentrations have been reported in newborns with confirmed necrotizing enterocolitis (NEC), there is substantial overlap between physiological and pathological values in preterm infants, particularly during the first weeks of life [4]. The clinical interpretation of FC remains a challenge, and longitudinal data describing its normal biological variability are still limited [4,5]. No studies have been conducted in Brazil.

Therefore, the primary objective of this prospective cohort study was to characterize the longitudinal variability of FC concentrations during the first month of life in preterm newborns admitted to a neonatal intensive care unit. Due to the incidence of confirmed NEC is relatively low in single-center cohorts, the evaluation of FC behavior in newborns with clinically suspected NEC was considered a secondary exploratory objective. Accordingly, the present study was not designed to assess the diagnostic performance of FC for NEC, but rather to improve the understanding of its longitudinal physiological variability in very preterm infants. By focusing on the natural longitudinal profile of FC in this vulnerable population, this study aims to contribute to a more appropriate interpretation of FC measurements in future neonatal research and clinical practice.

## 2. Subjects and Methods

### 2.1. Ethical Issues

This study involving human subjects, human material, and human data was conducted in accordance with the ethical principles established in the Declaration of Helsinki (1975, revised in 2013). In compliance with this declaration, the research protocol was reviewed and approved by the Ethics Committee of Hospital do Trabalhador prior to study initiation, ensuring adherence to both national and international ethical guidelines. Approval was granted under protocol number 3.368.353 on 4 June 2019. The legal guardians of all participants were informed about the study objectives and provided written informed consent before participation.

### 2.2. Participants

This prospective cohort study included preterm newborns up to 34 weeks of gestational age who were admitted to the NICUs of Hospital do Trabalhador in Curitiba, Paraná. This hospital serves patients from the public health system.

**Figure 1:**
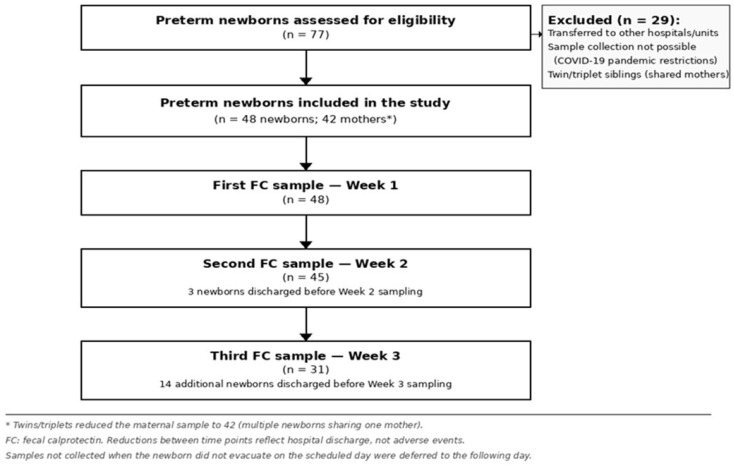
Flowchart of sample for fecal calprotectin measurement collected.

The assessments were conducted consecutively from August 2019 to March 2021, with a follow-up duration of up to 30 days or until hospital discharge, whichever occurred first. Inclusion criteria were consecutive preterm newborns up to 34 weeks of gestation born at the abovementioned NICU. Cases from other NICUs and those with critical heart disease or congenital anomalies were excluded. The sample comprised all eligible preterm newborns admitted to the study NICU during the enrollment period (convenience sample). Of the 77 newborns evaluated for eligibility, 48 were enrolled; Figure 1 illustrates the reasons for exclusion.

Newborns were classified as having clinically suspected NEC when they presented feeding intolerance (abdominal distension, vomiting or bloody stools) associated with radiological findings suggestive of intestinal involvement according to the institutional clinical protocol. Because the primary objective of this study was not to diagnose NEC, Bell staging was not prospectively applied. Therefore, these cases represent clinical suspicion rather than confirmed NEC and should be interpreted accordingly. Instead, cases were managed according to the institutional neonatal care protocol based on early clinical suspicion, frequently before fulfillment of standardized diagnostic criteria, reflecting the population admitted to the participating NICU during the study period, which may have introduced misclassification bias. Consequently, the subgroup analysis involving suspected NEC should be interpreted as exploratory rather than diagnostic.

The electronic and manual medical records of all included patients were reviewed; data were extracted and tabulated using Microsoft Excel^®^. For both mothers and preterm newborns, demographic, clinical, and laboratory data were collected. To minimize information bias, data extraction from medical records was conducted by two independent researchers. Discrepancies were resolved by consensus. All available clinical information was obtained from routine medical records, and no additional interventions or investigations were performed exclusively for research purposes. All samples were analyzed at the Immunopathology Laboratory of the Hospital de Clínicas, Curitiba, Brazil. Laboratory personnel were blinded to the participants’ clinical information throughout the analysis.

### 2.3. Fecal Calprotectin Measurement

Stool samples were collected from all the study patients on the seventh day of life. Subsequent collections were performed at 7-day intervals, resulting in approximately three samples per patient. If the newborn did not evacuate on day “seven,” the sample was collected on the next day. FC samples were collected according to a predefined weekly schedule, independent of clinical events. This sampling strategy was chosen to evaluate the longitudinal physiological behavior of FC during the neonatal period rather than its acute variation during episodes of gastrointestinal symptoms. In cases of suspected NEC, no additional event-triggered sampling was performed. Therefore, transient FC elevations occurring immediately before or during the onset of NEC-related symptoms may not have been captured. This sampling approach may have restricted the ability to capture acute changes in FC levels associated with NEC onset and may have underestimated peak values.

The fecal samples were stored at −20 °C until analysis. Before testing, the samples were thawed at room temperature. Calprotectin extraction was performed using 50 mg of stool and 2.5 g/mL of extraction buffer (B-CAL-EX). FC concentrations were measured using the commercial Bühlmann Calprotectin ELISA kit (Bühlmann Laboratories AG, Schönenbuch, Switzerland), a sandwich ELISA-based in vitro diagnostic assay for the quantitative measurement of calprotectin in human stool. This kit was selected on the basis of a literature review, due to its widespread use in published studies and strong reputation in the market. The laboratory professional performing the FC assays was blinded to the clinical status of the participants. Absorbance readings were obtained by spectrophotometry, and FC values were calculated accordingly. All samples were processed by the same trained laboratory professional throughout the study.

The following FC reference values were considered according to the manufacturer’s recommendations: levels < 50 µg/g are not indicative of gastrointestinal inflammation, levels 50–200 µg/g are considered indeterminate and may require repeat testing, and levels > 200 µg/g are indicative of organic disease with active gastrointestinal inflammation. Nevertheless, these reference values were originally established for adult populations and were adopted exclusively for descriptive purposes. Because no validated FC cutoff has been established for very preterm infants, all inferential analyses were primarily based on FC as a continuous variable, whereas categorical analyses using these thresholds should be interpreted with caution.

### 2.4. Statistical Analysis

Data were organized into frequency and contingency tables. Data normality was determined using the Shapiro–Wilk test. Continuous variables were reported as the median and interquartile range and analyzed using the Mann–Whitney test or Kruskal–Wallis test for nonparametric data. The Friedman test for paired samples was used to compare longitudinal FC concentrations across the three weekly measurements within each group. Comparisons between newborns with and without clinically suspected NEC at each time point were performed using the Mann–Whitney U test. Pearson’s correlation coefficient (r) was used to determine correlations between continuous variables. Categorical variables were expressed as percentages, and Fisher’s exact test and the chi-squared test were used for nonparametric and parametric data, respectively. Multivariate linear regression analysis including gestational age, birth weight, antibiotic use, and feeding type was performed; however, no considerable associations were identified. A *p* value of <0.05 was considered statistically significant. Statistical analyses were conducted using SPSS version 17.0 and GraphPad Prism version 6.0. This study was reported in accordance with the Strengthening the Reporting of Observational Studies in Epidemiology (STROBE) guidelines for cohort studies.

## 3. Results

During the study period, 124 stool samples were processed and analyzed, as depicted in the flowchart (Figure 1). Overall, 48 preterm newborns were prospectively examined, comprising 31 males and 17 females. The mean birth weight of the infants was 1212 g, and two-thirds (32 infants) were classified as extremely preterm newborns. Table 1 depicts the major clinical characteristics of the preterm newborns included in this study.

Among the 42 mothers included, 14.3% were aged <20 years, 61.9% were aged 20–34 years, and 23.8% were aged >35 years.

Regarding parity, 47.6% were primiparous. Vaginal delivery occurred in 30.9% of cases. No statistically significant association was identified between maternal characteristics and FC concentrations during the study period.

Overall, no statistically significant associations were detected between FC concentrations and the evaluated clinical characteristics (Table 2). Throughout longitudinal follow-up, FC concentrations exhibited marked interindividual and intraindividual variability. Median FC concentrations were 56 µg/g (IQR 35–139), 59 µg/g (IQR 34–136), and 65 µg/g (IQR 42–276) during the first, second, and third weeks of life, respectively. Although median FC values showed a slight numerical increase over time, no statistically significant longitudinal trend was identified.

Moreover, no significant association or correlation was observed between FC concentrations and variables such as time, birth weight, gestational age, type of diet, use of antibiotics or corticosteroids, and the need for resuscitation or mechanical ventilation during the study period (all *p* value > 0.05).

All newborns presenting FC concentrations above 200 µg/g at any time during follow-up were evaluated for NEC or other gastrointestinal complications. No confirmed NEC cases were identified, and elevated FC concentrations did not demonstrate a consistent temporal relationship with clinical gastrointestinal complications. 

A clinical suspicion of NEC was identified in five (10.4%) preterm newborns. The temporal relationship between symptom onset and FC sampling was heterogeneous because stool samples were collected according to the predefined study schedule rather than during acute clinical events. Consequently, FC measurements were obtained either before or after symptom onset in most cases. An exploratory review of newborns presenting elevated FC concentrations during follow-up did not reveal a consistent temporal relationship between FC elevation and clinically NEC.

When compared with newborns without clinically suspected NEC, no statistically significant differences in FC concentrations were identified, and no consistent discriminatory pattern was observed between groups. However, given the small number of newborns with clinically suspected NEC and the absence of confirmed NEC cases, these subgroup findings should be interpreted as exploratory rather than confirmatory.

**Figure 2:**
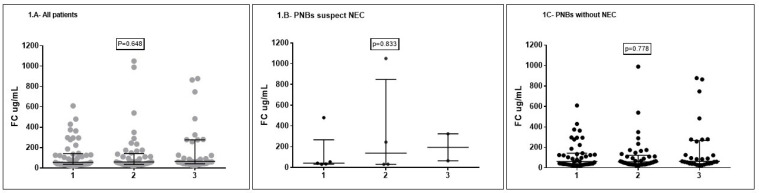
Fecal Calprotectin levels in all examined patients (**1A**), in patients with suspected NEC (**1B**), and in those without clinical suspicion of NEC (**1C**).

Figure 2 illustrates the FC levels in all examined patients (Figure 2(1A)), in patients with suspected NEC (Figure 2(1B)), and in those without clinical suspicion of NEC (Figure 2(1C)). No cases progressed to death during the study period.

Multivariable models adjusted for potential confounders showed no statistically significant associations between the outcome and gestational age subgroup, feeding type, postnatal age trajectory, or antibiotic exposure (all *p* > 0.05). Similarly, maternal and perinatal factors, including mode of delivery, antenatal corticosteroid exposure, maternal chorioamnionitis, and probiotic use, were not significantly associated with the outcome after adjustment for potential confounders (all *p* > 0.05).

## 4. Discussion

This prospective cohort study primarily characterized the longitudinal behavior of fecal calprotectin (FC) concentrations during the first month of life in preterm newborns. The principal finding was the substantial intraindividual and interindividual variability of FC levels, with no consistent temporal pattern throughout follow-up. These findings reinforce the concept that FC exhibits dynamic physiological behavior during the neonatal period and highlight the challenges of interpreting isolated FC measurements in very preterm infants. Because no confirmed NEC cases occurred during the study period, the present results should be interpreted primarily as describing the baseline biological variability of FC rather than its diagnostic performance for necrotizing enterocolitis.

Although thresholds have been proposed for FC in symptomatic preterm infants, such as 363 µg/g for mild enteropathy and 636 µg/g for severe enteropathy [6], FC levels in preterm newborns are affected by multiple factors, particularly postnatal age and intestinal immaturity. The marked variability observed in our cohort is consistent with previous evidence indicating that FC levels fluctuate considerably during the first month of life [7].

In adult and pediatric populations, FC levels ≥ 100 µg/g are commonly associated with intestinal inflammation. Conversely, preterm newborns may present increased FC levels even in the absence of overt disease. The suggested cutoff values for infants vary widely, with levels such as 538 µg/g proposed for infants aged 1–6 months [7]. These observations emphasize the limitations of applying adult-derived thresholds to neonatal populations. In our study, FC levels were primarily analyzed as a continuous variable, and any interpretation based on categorical thresholds must be considered exploratory.

The sampling strategy probably contributed to the variability observed in our study. Stool samples were collected at 7-day intervals, which may not adequately capture the rapid fluctuations associated with acute conditions such as NEC. Because sampling followed a predefined schedule, FC measurements were not temporally aligned with symptom onset, restricting the characterization of pre-, during-, and post-symptom trajectories. This methodological aspect should be considered when interpreting the absence of a consistent temporal relationship between FC concentrations and clinically suspected NEC in the present cohort. Financial constraints influenced the decision to collect a limited number of samples per patient. In a resource-limited setting within a middle-income country, conducting research imposes budgetary restrictions limiting the feasibility of more intensive longitudinal sampling strategies. Moreover, the irregular bowel habits of preterm newborns frequently precluded sample collection during clinically relevant periods, potentially causing missed peaks of FC levels. Current evidence does not establish an optimal frequency for FC monitoring in preterm populations [5]. Even in studies using more frequent sampling, FC alone has not been demonstrated to reliably function as a diagnostic marker for NEC [8]. Furthermore, fecal samples are inherently heterogeneous, and variability may be mitigated by averaging measurements from multiple samples [9]. Accordingly, our findings should not be interpreted as evidence against a biological association between FC and intestinal inflammation, but rather as illustrating the limitations of scheduled isolated sampling for evaluating an inherently dynamic biomarker.

Among the five newborns with clinically suspected NEC, FC concentrations demonstrated heterogeneous trajectories before and after symptom onset, without a consistent discriminatory pattern. However, interpretation of these findings should be made cautiously because stool sampling was not synchronized with clinical presentation, no cases fulfilled criteria for confirmed NEC, and the exact temporal interval between symptom onset and FC measurement was not prospectively recorded. Consequently, this subgroup analysis should be regarded as exploratory and should not be interpreted as an evaluation of the diagnostic accuracy of FC.

A meta-analysis published in 2020 reported pooled sensitivity and specificity values of 86% and 79%, respectively, for FC in diagnosing NEC [10]. However, these findings remain controversial, as subsequent studies have yielded inconsistent results. In particular, FC levels have not reliably differentiated between preterm newborns who develop enteropathies and those who do not, or between controls and those who progress to more advanced stages of NEC (Bell stages II or III) [11,12]. Similarly, Campeotto et al. did not identify a prognostic value for FC in enteropathy in a cohort of 121 neonates, potentially due to the substantial intraindividual and interindividual variability observed in very preterm infants [11]. Furthermore, Yan-Qiu Xie et al. demonstrated that FC levels are affected by multiple factors across different postnatal periods and suggest that FC should not be interpreted in isolation but may be considered a complementary biomarker along with clinical and laboratory parameters [13].

The increased FC levels observed in preterm newborns probably reflect the increased migration of neutrophils and macrophages into the intestinal lumen, possibly related to increased intestinal permeability and mucosal immaturity. Whether this increase represents a physiological phenomenon or a pathological response remains uncertain [14]. Moreover, the relationship between perinatal factors and FC levels is not completely established and may be affected by variables such as stress, pain, and feeding practices [15]. This study identified no significant associations between FC concentrations and neonatal variables, including weight, gestational age, feeding type, and the use of antibiotics and corticosteroids. Other perinatal factors potentially associated with FC concentrations, such as antenatal corticosteroid exposure, maternal chorioamnionitis, probiotic administration, and mode of delivery, have been reported in previous studies [15]. These variables were not prospectively collected in a standardized manner and therefore could not be included in the multivariable analysis. Their potential influence on FC variability cannot be excluded and should be explored in future prospective studies.

An additional consideration is the definition of clinically suspected NEC. Although the Bell classification is widely accepted for staging NEC, it was not systematically applied in this study because the primary objective was not to establish a diagnosis of NEC but to characterize the longitudinal variability of FC concentrations. In routine neonatal practice, infants presenting with early signs of intestinal compromise, such as abdominal distension and clinical deterioration, are frequently managed as suspected NEC before fulfilling standardized diagnostic criteria, with the aim of preventing progression to advanced disease.

FC use as a biomarker is well established in inflammatory bowel disease across pediatric and adult populations, with clearly defined cutoff values and widespread clinical applicability [16,17]. However, its role in preterm newborns remains uncertain. Considering the high morbidity and mortality associated with NEC, early clinical intervention is often prioritized in cases suggesting intestinal inflammation. Although this approach may improve outcomes, it may also impact biomarker interpretation and contribute to variability in FC levels.

Nonetheless, several limitations must be acknowledged, including the small sample size, the single-center design, and the absence of confirmed NEC cases. Consequently, the number of clinically suspected cases was limited, and the study may be underpowered to clinically detect notable associations. The predominance of extremely preterm newborns may have affected FC levels because of greater intestinal immaturity. The temporal discrepancy between sample collection and symptom onset represents another important limitation. Moreover, individual-level clinical data for the five preterm newborns with suspected NEC were not systematically collected within the scope of this study. Future studies should prospectively document individual clinical outcomes using standardized criteria such as Bell staging to enable more relevant biomarker analyses.

Taken together, our findings reinforce the concept that FC concentrations exhibit marked physiological variability during early postnatal life, limiting the interpretation of isolated measurements in preterm newborns. Rather than refuting a potential role for FC in NEC, the present study highlights the importance of considering sampling strategy, clinical timing, and neonatal physiological maturation when interpreting FC concentrations.

In conclusion, this prospective cohort demonstrated substantial longitudinal intraindividual and interindividual variability of FC concentrations during the first month of life in preterm newborns. These results emphasize that FC should be interpreted cautiously in very preterm infants and support future multicenter studies incorporating standardized NEC definitions and event-driven serial sampling to better define the complementary role of FC as a neonatal biomarker.

## Figures and Tables

**Table 1:** Clinical characteristics of the preterm newborns included in the study.

Data from Newborns (*n* = 48)	*n*	%
Sex		
Female	17	35.4
Male	31	64.6
Weight classification		
Low-birth-weight newborn	11	22.9
Very-low-birth-weight newborn	24	50.0
Extreme-low-birth-weight newborn	13	27.1
Classification according to gestational age		
Very preterm newborn	16	33.4
Extremely preterm newborn	32	66.7
Weight and gestational age correlation		
Appropriate for gestational age	29	60.4
Small for gestational age	19	39.6
Need for neonatal resuscitation	39	81.3
Continuous positive airway pressure	16	33.3
Intubation in neonatal resuscitation	22	45.8
Umbilical catheters	44	91.7
Peripherally inserted central catheters	45	93.8
Transfusion need	21	43.8
Phototherapy need	32	66.7
Pulmonary surfactant need	44	91.7
Parenteral nutrition need	43	89.6
Start of feeding		
1st day	20	43.5
2nd day	22	47.8
3rd day or more	4	8.7
Use of breast milk	47	97.9
Antibiotic use	38	80.9
Use of antibiotics for more than ten days	13	30.2
Use of corticosteroids after birth	29	60.4
Need for mechanical ventilation	35	72.9
Suspected necrotizing enterocolitis	5	10.4

**Table 2:** Fecal calprotectin levels (µg/g) across three weekly time points in preterm newborns, overall and stratified by clinical suspicion of NEC.

	*n*	Median	IQR	*n*	Median	IQR	*n*	Median	IQR	*p* Value (Friedman Test)
	Week 1			Week 2			Week 3			
Total patients	48	56	(35.0–139.0)	45	59	(34.0–136.0)	31	65	(42.0–276.0)	0.648
Suspected cases of NEC										
No	43	62	(35.0–143.0)	41	59	(35.0–112.0)	29	64	(42.0–258.0)	0.788
Yes	5	42	(34.0–55.0)	4	139	(31.5–647.5)	2	195	(65.0–325.0)	0.833

Columns represent Week 1 (*n* = 48), Week 2 (*n* = 45), and Week 3 (*n* = 31). Values are expressed as median (IQR). The *p*-values shown in the last column correspond exclusively to within-group longitudinal comparisons across the three time points using the Friedman test. Between-group comparisons (clinically suspected NEC vs. no clinically suspected NEC) at each time point were performed using the Mann–Whitney U test and showed no statistically significant differences. Because of the small number of newborns with clinically suspected NEC, subgroup analyses should be interpreted as exploratory. IQR: Interquartile range; NEC: Necrotizing enterocolitis. *p*-values for total patients and each NEC subgroup reflect within-group longitudinal comparisons (Friedman test). Between-group comparisons (Yes vs. No) were performed using Mann–Whitney test.

## Data Availability

The original contributions presented in this study are included in the article. Further inquiries can be directed to the corresponding author.
